# Self-Assembly of Diboronic Esters with U-Shaped Bipyridines: “Plug-in-Socket” Assemblies

**DOI:** 10.1021/acs.cgd.1c00382

**Published:** 2021-07-13

**Authors:** Christopher J. Hartwick, Shweta P. Yelgaonkar, Eric W. Reinheimer, Gonzalo Campillo-Alvarado, Leonard R. MacGillivray

**Affiliations:** † Department of Chemistry, University of Iowa, Iowa City, Iowa 52242, United States; ‡ Rigaku Oxford Diffraction, 9009 New Trails Drive, The Woodlands, Texas 77381, United States

## Abstract

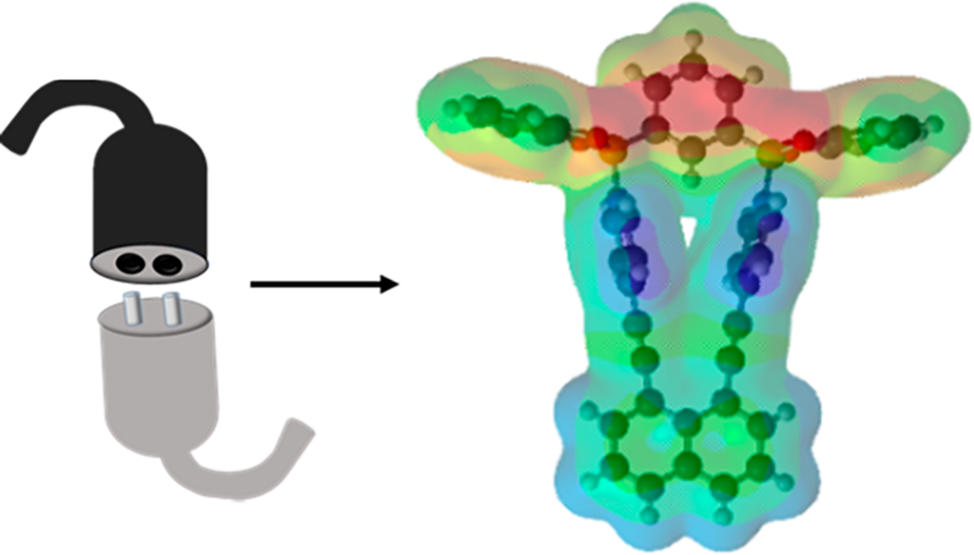

Self-assembled complexes utilizing the ditopic dative bond acceptor 1,3-diboronic acid with catechol and complementary U-shaped donors in the form of 1,8-dipyridylnaphthalenes (1,8-bis(4-pyridyl)naphthalene (**DPN**), 1,8-bis(4-ethylenylpyridyl)naphthalene (**DEPN**), and 1,8-bis(4-ethynylpyridyl)naphthalene (**DAPN**)) yielded discrete two-component structures. The assemblies exhibit “plug-in-socket” geometries. DFT calculations are consistent with the donor pyridyl and acceptor catecholate being electron poor and rich, respectively. The assemblies pack via π–π interactions and support the inclusion of a solvent (i.e., **DPN**, **DAPN**). The materials may form a basis for the design of complex B-based structures (e.g., supramolecular dyads).

## Abstract 2

Self-assembly of U-shaped bipyridines and a diboronic ester affords “plug-in-socket” arrays.

## Introduction

Self-assembly processes involving diboronic esters are increasingly prevalent in the design of complex supramolecular assemblies and architectures.^1^ Linear diboronic acids on crystallization with di- and tritopic pyridines as linkers, for example, have generated macrocycles and cages, respectively. The materials, which also exhibit propensities to include solvent guests (e.g., aromatics) within and exterior to the self-assembled structures, are promising for applications in areas such as separations, sensing, and electronics.^2−5^ While noncovalent bonds such as π–π interactions, hydrogen bonding, and electrostatics have been more traditionally useful to create self-assembled structures, B←N coordination involving pyridine linkers has more recently received widespread attention. The pyridine linkers to date have been based on divergent geometries (e.g., 4,4′-bipyridine), many of which draw inspiration from studies that aim to generate hydrogen-bond and metal-mediated frameworks and solids.

U-shaped heterocyclic aza-aromatics based on the 1,8-disubstitution of naphthalenes (**1,8-nap**) are useful constructs in the field of molecular recognition.^6^ The hydrogen-bonding and metal-coordination capabilities of the 1,8-diacridines have been exploited for enantioselective and fluorescence sensing of chiral carboxylic acids and metal ions, respectively.^7,8^ More recently, hydrogen bonding and coordination involving the **1,8-nap** framework has facilitated inter- and intramolecular photocyclizations in the solid state.^9,10^ While the assembly properties of boronic acids can be expected to enhance the structural chemistry of members of the **1,8-nap** framework, derivatives of **1,8-nap** have not been applied to self-assembly using B←N bonding. Indeed, members of the **1,8-nap** family of molecules are becoming more synthetically accessible, given the amenability of the bipyridines to be synthesized by mainstream cross-coupling reactions.

**Scheme 1:**
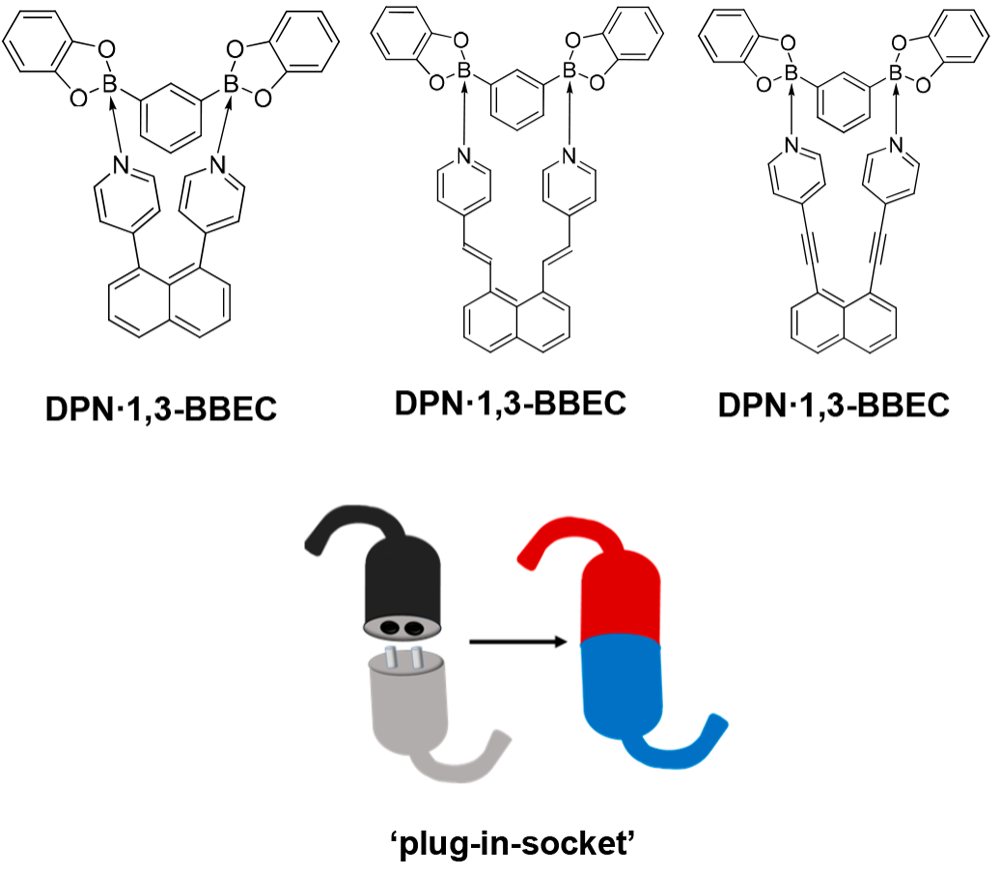
Structural Representations of the **1,8-nap** Assemblies (Top) and Iconic View (Bottom)

Herein we describe the self-assembly of a series of 1,8-dipyridylnaphthalenes with diboronic esters of 1,3-catecholates. Specifically, a combination of 1,8-bis(4-pyridyl)naphthalene (**DPN**), 1,8-bis(4-ethylenylpyridyl)naphthalene (**DEPN**), and 1,8-bis(4-ethynylpyridyl)naphthalene (**DAPN**) with 1,3-benzenediboronic acid catechol diester (**1,3-BBEC**) affords the discrete 1:1 assemblies **DPN**·**1,3-BBEC**, **DEPN**·**1,3-BBEC**, and **DAPN**·**1,3-BBEC**, respectively. In all cases, the bipyridines interact with the diboron building unit akin to a macroscopic “plug-in-socket” array (Scheme 1). For **DPN** and **DAPN**, the plug-in-socket arrays generate the host–guest solids **DPN**·**1,3-BBEC**·CHCl_3_ and **DAPN**·**1,3-BBEC**·2(*m*-xylene). We expect the U-shaped donors described here to be viable building blocks for the construction of complex B-based assemblies sustained by B←N bonding.

## Experimental Section

Toluene, chloroform, and catechol were purchased from Millipore-Sigma and used as received. Benzene-1,3-diboronic acid was purchased from Oakwood Laboratories and used without further purification. **DPN**, **DEPN**, and **DAPN** were synthesized according to the literature.^9,11,12^ The formation of each assembly was accomplished by forming chloroform solutions of the corresponding bipyridine, 1,3-benzenediboronic acid, and catechol (ratio 1:1:2) and subsequently placing either toluene (**DPN** and **DEPN**) or *m*-xylene (**DAPN**) in a loosely secured screw-capped vial. Slow solvent evaporation resulted in diffraction-quality crystals of the adducts within 2 days. Crystal structures were solved using Olex2.^13^ Density functional theory (DFT) calculations (B3LYP/6-31G* level) were conducted using Spartan 18.^14^

### X-ray diffraction

Single crystals suitable for X-ray diffraction analyses were secured to X-ray transparent magnetic mounts using Paratone oil and mounted on a Bruker D8 Venture diffractometer with a Photon III detector. All single-crystal measurements were performed at 150 or 190 K or at room temperature using either Cu Kα (λ = 1.54184 Å) or Mo Kα (λ = 0.71073 Å) radiation.

## Results and Discussion

**Scheme 2:**
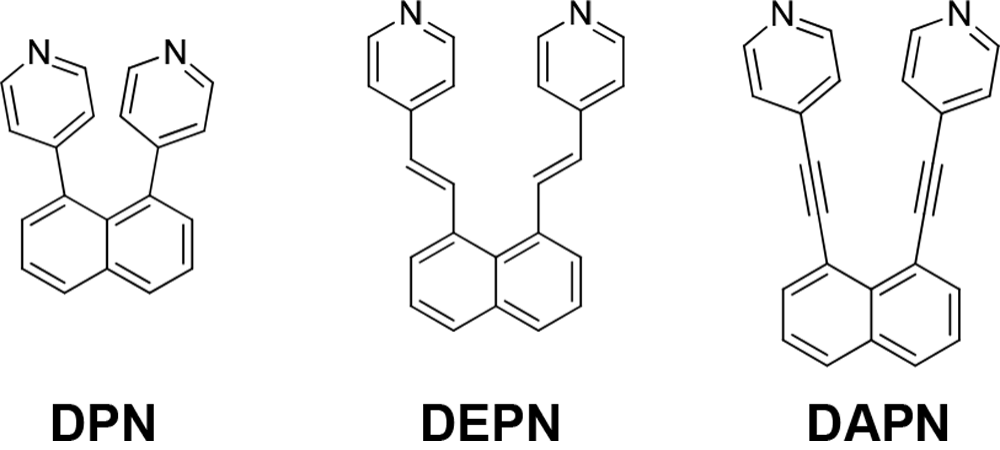
Bipyridines **DPN**, **DEPN**, and **DAPN**

**DPN**, **DEPN**, and **DAPN** are attractive owing to the cofacial geometries of the 4-pyridyl groups (Scheme 2). The pyridyls are generally twisted nearly perpendicular to the naphthalene ring system. The cofacial geometry has been exploited by Wolf for molecular recognition and enantiosensing (e.g., amino acids).^7,15^ Interactions involving the N atoms can facilitate complexation with organohalides, hydrogen bonding, and metal complexation. We are unaware of examples wherein the bipyridines have been studied in the context of B←N coordination.

### X-ray Structures of Individual Components

**Figure 1:**
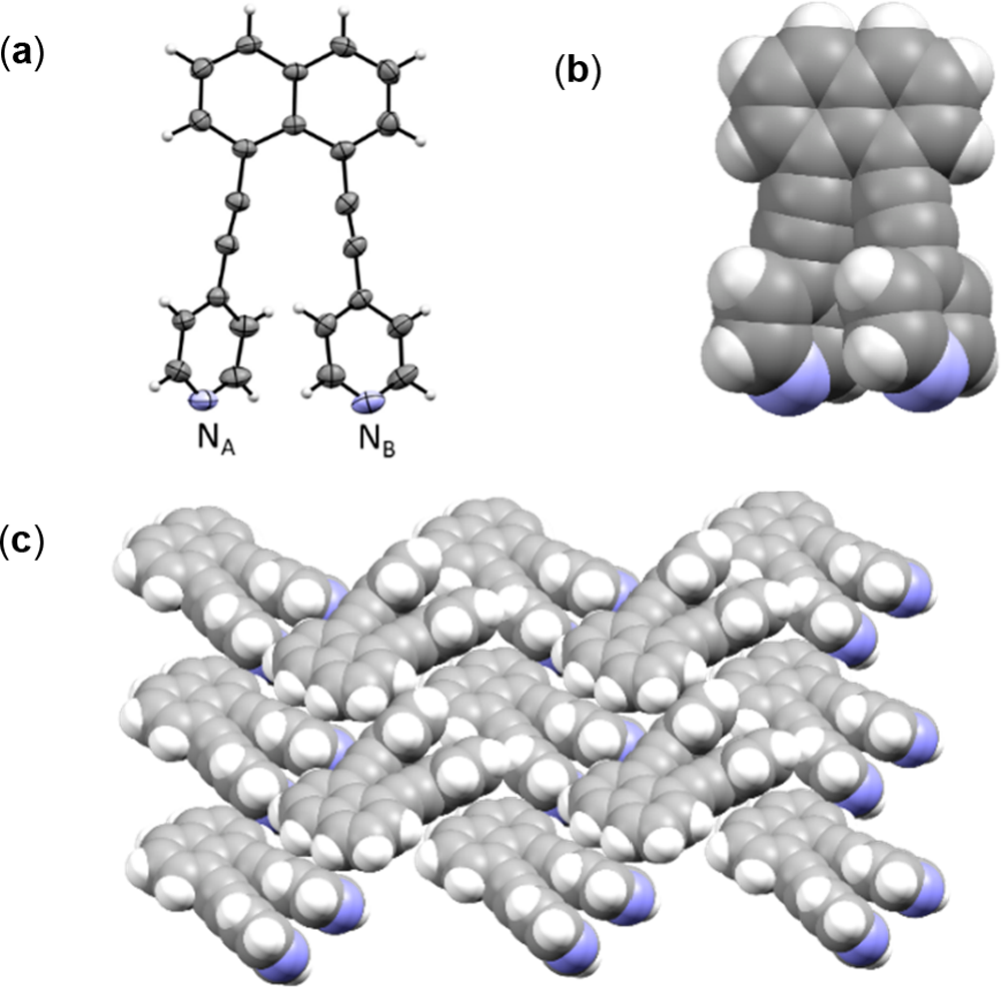
X-ray structure of **DAPN**: (a) ORTEP diagram; (**b**) space-filling representation; (c) 2D packing.

The crystal structures of two polymorphs of **DPN** have been reported,^16^ and we have described the structure of **DEPN**.^9^ The X-ray structure of **DAPN** is described here for the first time. **DAPN** crystallizes in the chiral orthorhombic space group *P*2_1_2_1_2_1_ with one full molecule in the asymmetric unit (Figure 1 and Table 1). Both 4-pyridyl groups are twisted from coplanarity (36.7, 49.1°) with respect to the naphthyl rings (Figure 1a and Table 2). The pyridyl rings are oriented approximately cofacially (19.7°) (Figure 1b). The N atoms of the stacked pyridyl rings are separated at a distance (4.49 Å) greater and less than those of **DPN** and **DEPN**, respectively. The molecule forms a 2D layered structure with adjacent bipyridines interacting via edge-to-face C–H···π forces (C–H···centroid 3.73 Å) (Figure 1c).

**Table 1:** Crystallographic Data for **DAPN** and **1,3-BBEC**

compound	DAPN	1,3-BBEC
CCDC code	2074336	2074338
formula	C_24_H_14_N_2_	C_18_H_12_B_2_O_4_
formula wt	330.37	313.90
temp (K)	190(2)	297(2)
space group	*P*2_1_2_1_2_1_	*P*2_1_2_1_2
*a* (Å)	9.0979(9)	22.542(2)
*b* (Å)	11.7870(12)	4.7936(5)
*c* (Å)	16.0856(16)	6.9265(7)
α (deg)	90	90
β (deg)	90	90
γ (deg)	90	90
*V* (Å^3^)	1725.0(3)	748.46(13)
*Z*	4	2
calcd density (g/cm^3^)	1.272	1.393
μ (mm^–1^)	0.075	0.096
scan	ω and φ scans	ω and φ scans
θ range for data collection (deg)	2.532–27.918	2.941–26.340
no. of rflns measd	35632	12894
no. of indep obsd rflns	4109	1522
no. of indep rflns (*I* > 2σ)	3070	1313
no. of data/restraints/params	4109/0/235	1522/0/110
*R*_int_	0.0466	0.0538
final *R* indices (*I* > 2σ)	0.0444	0.0319
R indices (all data)	0.0713	0.0403
goodness of fit on *F*^2^	1.057	1.045

**Table 2:** Structural Metrics for **DPN**, **DEPN**, and **DAPN**

	**DPN**	**DEPN**	**DAPN**
N···N (Å)	4.03 (form I), 3.99 (form II)	5.55, 5.15	4.49
pyridyl twist (deg)	59.0, 51.4 (form I); 69.2, 68.5 (form II)	49.4, 52.3; 50.2, 40.8	36.7, 49.1

**Figure 2:**
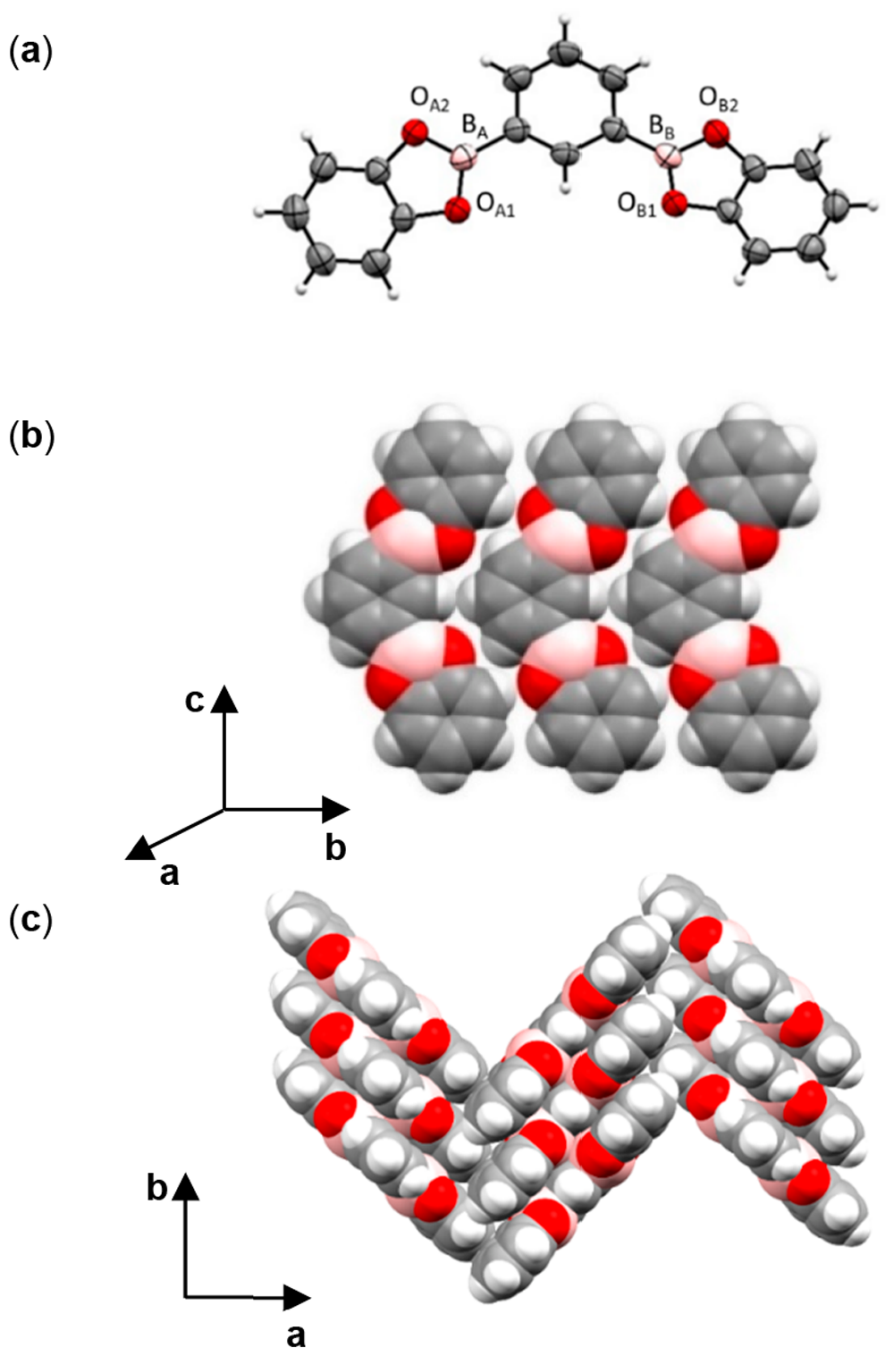
X-ray structure **1,3-BBEC**: (a) ORTEP diagram; (b) head-to-tail assembly; (c) herringbone packing.

The diboronic ester **1,3-BBEC** crystallizes in the monoclinic space group *P*2_1_2_1_2 with half of a molecule in the asymmetric unit (Figure 2). The terminal catecholate groups are twisted slightly (7.35°) from the central aromatic ring system (Figure 2a). The molecule self-assembles head-to-tail along the *c* axis, being sustained by C–H···O hydrogen bonds (Figure 2b). **1,3-BBEC** packs in a herringbone arrangement within the crystallographic *ab* plane (Figure 2c).

### Plug-in-Socket Assemblies

**Figure 3:**
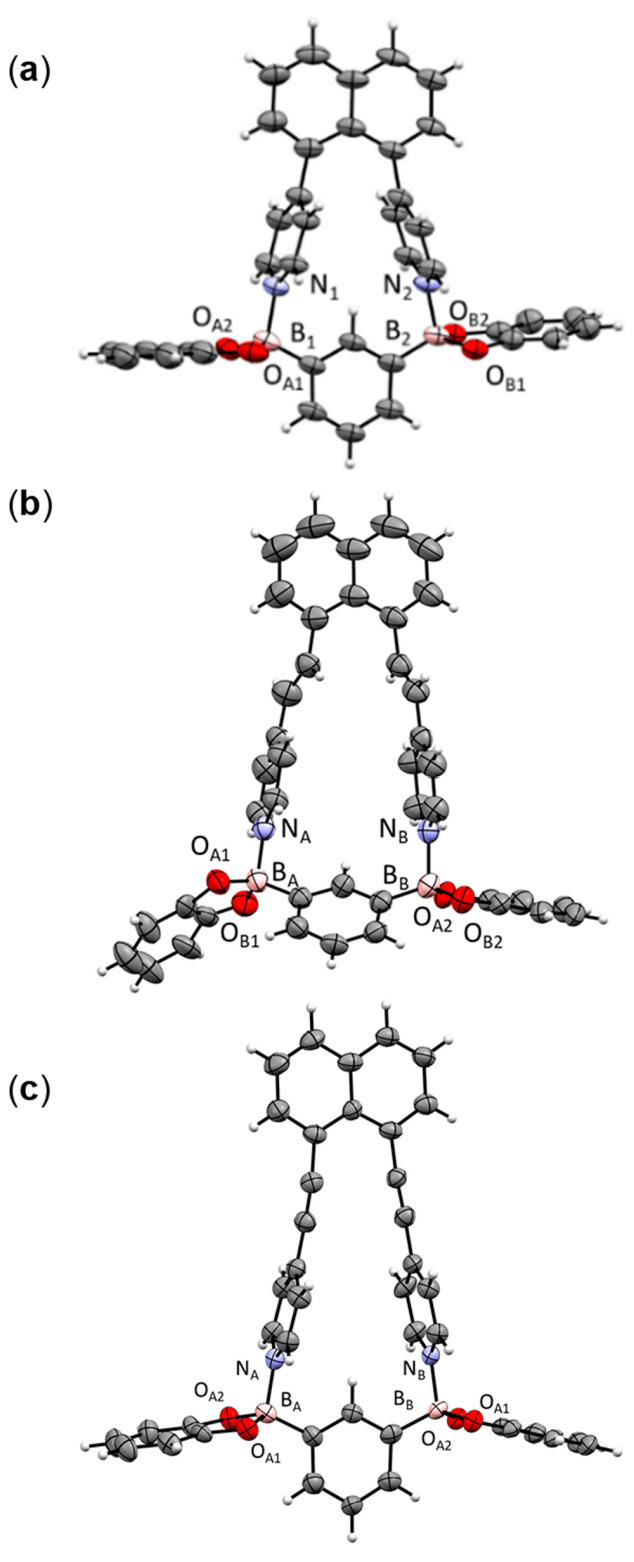
X-ray structures of (a) **DPN·1,3-BBEC**; (b) **DEPN·1,3-BBEC**; and (c) **DAPN·1,3-BBEC**.

For **DPN**, **DEPN**, and **DAPN**, the self-assembly process involving **1,3-BBEC** affords two-component complexes with structures that conform to “plug-in-socket” types of assemblies (Figure 3). The N atoms of the pyridyl groups of each array adopt an approximate parallel orientation and engage in B←N coordination (Table 3). The B–N bond distances are generally comparable and are only slightly longer than those of reported 4-pyridyl-based assemblies and macrocycles.^9^ The coordination to the B atoms generally results in an increase to the N···N distances relative to those of the pure bipyridines. The greater N···N distance is associated with the flexible, or “rotatable”, C=C group of **DEPN**.^9^ The B···B distances of the assemblies are also generally larger versus that of pure **1,3-BBEC** (Tables 2 and 3).

**Table 3:** Structural Metrics for **1,3-BBEC** Assemblies of **DPN**, **DEPN**, and **DAPN**

	**DPN·1,3-BBEC**	**DEPN·1,3-BBEC**	**DAPN·1,3-BBEC**
B–N (Å)	1.6688(2), 1.6480(2)	1.6530(2), 1.6752(2)	1.643(8), 1.643(8); 1.635(8), 1.648(8)
N_1_–N_2_ (Å)	4.62(1)	5.116(5)	4.857(7), 4.888(7)
B···B (Å)	5.29(1)	5.25(1)	5.31(1), 5.29(1)
twist from orthogonality (α_1_, α_2_) (deg)	63.82(3), 59.76(3)	59.07(8), 55.17(8)	109.4(2), 103.8(2), 98.7(2), 102.0(2)

The greater distances to the N atoms afforded by alkenyl and alkynyl groups give rise to pyridyl twist angles that deviate from orthogonality. **DPN·1,3-BBEC** and **DEPN·1,3-BBEC** exhibit twist angles <90°, while **DAPN·1,3-BBEC** exhibits a twist angle >90°. Two of the three complexes based on **1,3-BBEC** are solvent inclusion compounds.^17,18^ In general, the shapes of the complexes are based on aromatic ring systems that alternate approximately perpendicularly to each other.

**Figure 4:**
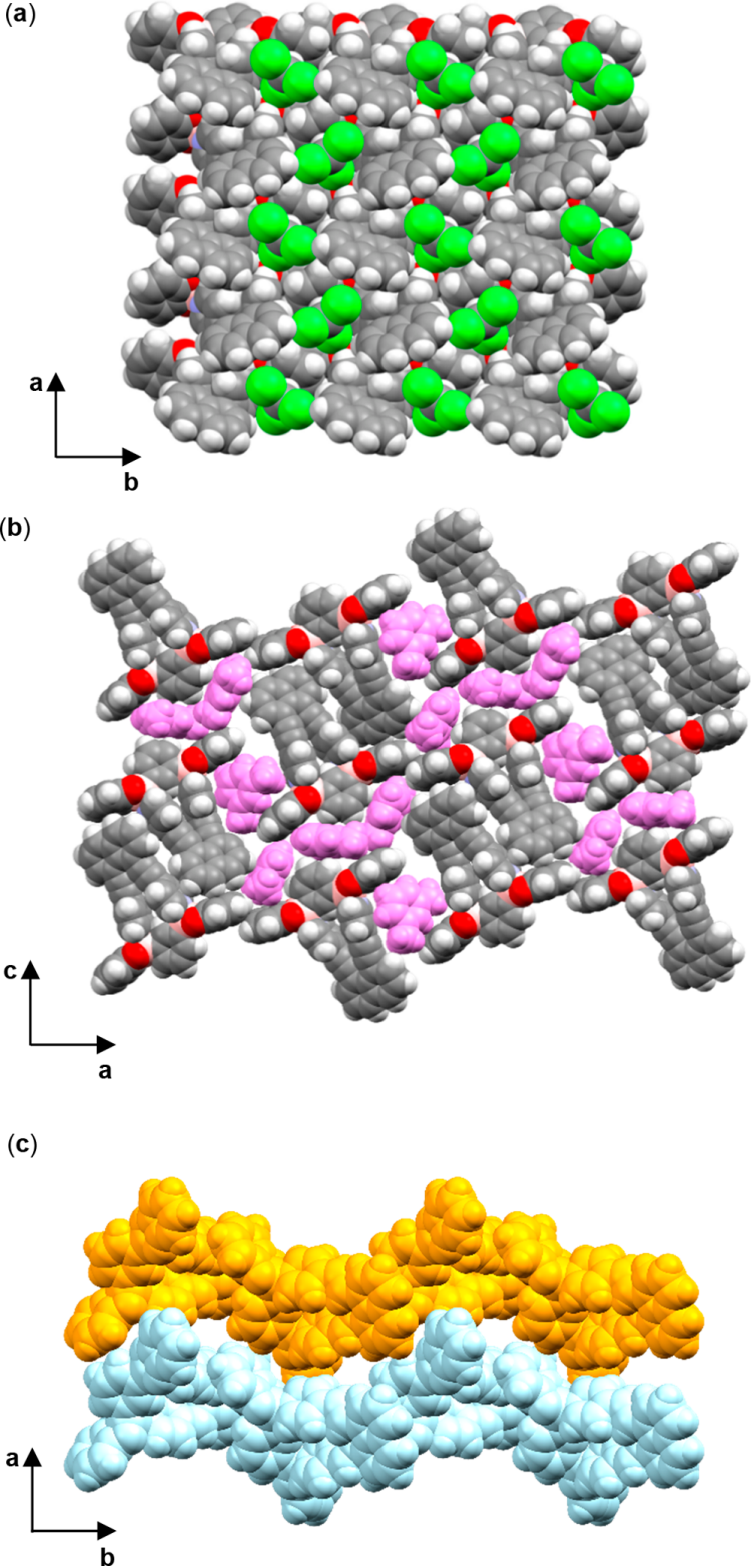
X-ray structures: (a) **DPN·1,3-BBEC**; (b) *m*-xylene inclusion (purple) of **DAPN·1,3-BBEC**; (c) undulating layers of **DEPN·1,3-BBEC**.

**DPN·1,3-BBEC** crystallizes with one full complex and one CHCl_3_ molecule in the asymmetric unit. A single catecholate ring lies disordered (occupancies: 50:50), as does the chloroform molecule (occupancies: 70:30). The included solvent is nestled adjacent to the B←N linkage. The Cl atoms participate in Cl···O interactions with the catecholate and C atoms of the pyridyl group. The complexes and solvent molecules form 2D layers within the *ab* plane (Figure 4a). The packing is manifested such that the naphthyl units point in the same direction along the *c* axis. Several π–π interactions define the packing of the pyridyl edges with the central phenyl ring faces of **1,3-BBEC** (edge···centroid = 3.60 Å) and pyridyl edges with naphthyl faces (edge···centroid = 3.51 Å) (Table 4). The catechol rings display face-to-face π–π stacking of adjacent complexes (centroid··· centroid = 3.82 Å), while the central phenyl edges and naphthyl ring faces also exhibit edge-to-face interactions (edge···centroid = 3.63 Å).

**Table 4:** Crystallographic Data for **DAPN** and **1,3-BBEC**

	**DPN·1,3-BBEC**·CHCl_3_	**DEPN·1,3-BBEC**	**DAPN·1,3-BBEC**·2(*m*-xylenes)
CCDC code	2074340	2074337	2074339
formula	C_39_H_27_B_2_Cl_3_N_2_O_4_	C_42_H_30_B_2_N_2_O_4_	C_58_H_46_B_2_N_2_O_4_
formula wt	715.62	648.30	856.59
temp (K)	150(2)	298(2)	150(2)
space group	*Pca*2_1_	*P*2_1_/*c*	*Cc*
*a* (Å)	14.1949(19)	10.5142(12)	32.765(4)
*b* (Å)	13.2800(19)	22.877(3)	10.6210(12)
*c* (Å)	17.799(4)	14.0824(16)	27.323(3)
α (deg)	90	90	90
β (deg)	90	96.813(3)	96.369(5)
γ (deg)	90	90	90
*V* (Å^3^)	3355.3(10)	3363.4(7)	9449.6(19)
Z	4	4	8
calcd density (g/cm^3^)	1.417	1.280	1.204
μ (mm^–1^)	2.848	0.081	0.074
scan	ω and φ scans	ω and φ scans	ω and φ scans
θ range for data collection (deg)	4.559–67.068	2.144–26.420	2.017–26.447
no. of rflns measd	39573	66210	72523
no. of indep obsd rflns	5406	6905	19034
no. of indep rflns (*I* > 2σ)	4449	4175	12068
no. of data/restraints/params	5406/73/520	6905/78/483	19034/8/1198
*R*_int_	0.0807	0.0701	0.0763
final *R* indices (*I* > 2σ)	0.0658	0.0537	0.0762
*R* indices (all data)	0.0763	0.0993	0.1198
goodness of fit on *F*^2^	1.082	1.025	1.009

**DAPN·1,3-BBEC** crystallizes with two full complexes and four *m*-xylene molecules in the asymmetric unit. The complexes form edge-to-face dimers (Figure 4b). Edge-to-face packing between a pyridyl group and central phenyl ring forms T-shaped motifs (C–H···centroid = 3.50 Å) that alternate to form chains. Due to π–π interactions and the packing of the complexes, the included *m*-xylene molecules exhibit edge-to-face interactions with the assemblies. Being effectively pinched between the naphthyl adduct (edge···centroid = 3.85 Å) and central phenyl rings (edge···centroid = 3.73 Å), one molecule of *m*-xylene interacts by edge-to-face forces. The central phenyl ring edges of the assemblies similarly fit with one *m*-xylene between the complex edges (edge···centroid = 3.69 Å). The catecholate rings are also effectively sandwiched orthogonally with *m*-xylene molecule (edge···centroid = 3.53 Å).

**DEPN·1,3-BBEC** crystallizes with one full complex in the asymmetric unit. A single catecholate ring lies disordered (occupancies: 50:50). The complex self-assembles to form a packing arrangement sustained by edge-to-face interactions involving the pyridyl and naphthyl ring systems similarly to **DAPN·1,3-BBEC** (edge···centroid = 3.70 Å). Undulating layers of the packed complexes are present in the crystallographic *bc* plane resulting from twisting of adducts (59.2°) (Figure 4c).

### B-Based Assemblies

While there have been several reports of discrete assemblies sustained by B←N linkages, we are unaware of a discrete B-based assembly involving the 1,8-naphthyl geometry.^19^ The U-shaped scaffold has been used in the assembly of Ag(I) ions, which are separated at distances shorter (Ag···Ag distance = 3.45–3.80 Å) than for the B atoms of **DPN·1,3-BBEC** (5.29 Å), **DEPN·1,3-BBEC** (5.25 Å), and **DAPN·1,3-BBEC** (5.31, 5.29 Å). The N···N distances of the Ag(I) complexes^9^ (3.79–4.21 Å) are shorter than those of the B complex (Table 2). Hydrogen^10^ (3.77–3.96 Å)- and halogen-bonded^11^ (4.20 Å) cocrystals of **DPN** have also reported with N···N distances that exhibit similar separations. The U-shaped bipyridines of the cocrystals exhibit shorter distances in comparison to the B complexes. Extended assemblies based on B←N linkages have also been reported. Work by Severin involving **1,3-BBEC** shows the efficacy of B←N linkages to form networks^20^ with regenerative abilities. Utilizing an array of pyridyl- and imidazolyl-functionalized molecules, a wide range of architectures for self-assembly involving the B←N bond were realized.^20−23^

**Figure 5:**
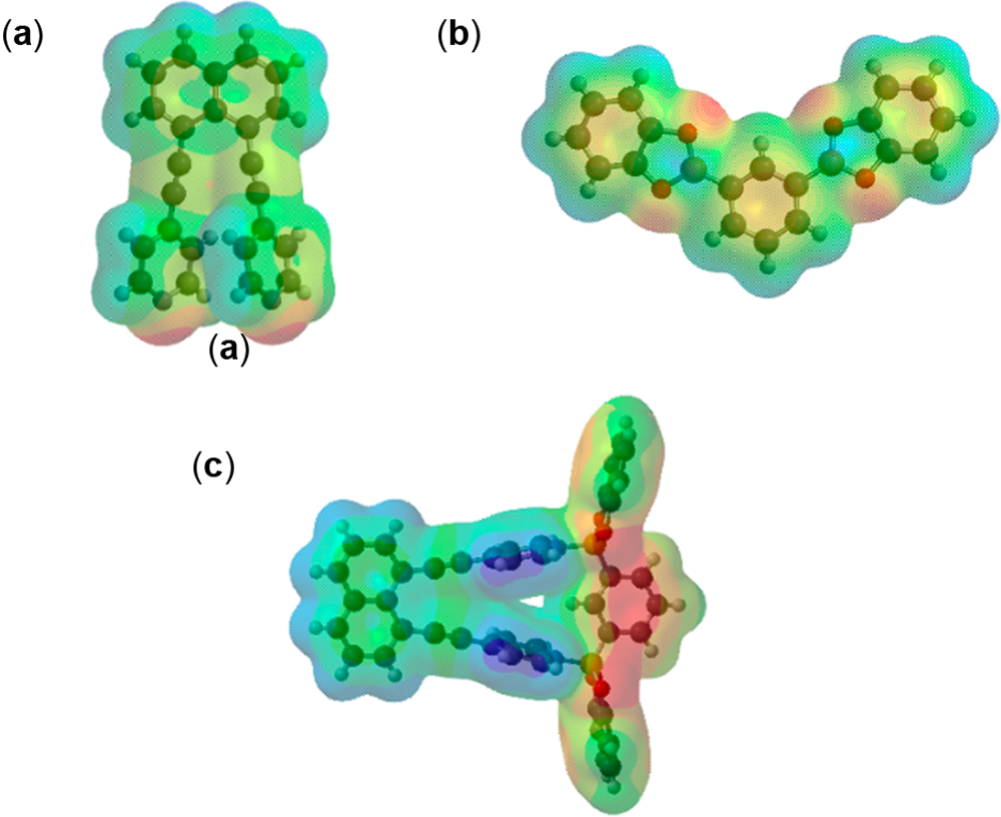
DFT-calculated structures: (a) **DAPN**; (b) **1,3-BBEC**; (c) **DAPN·1,3-BBEC**. Color code: blue, positive electrostatic charge; red, negative electrostatic charge.

DFT calculations indicate an increase in electropositivity of the coordinated pyridyl groups (Figure 5).^14^ The ranges of the electrostatic potential values (kJ/mol) for the pyridyl groups are **DAPN** (80–82) > **DPN** (71–73) > **DEPN** (56–58) (i.e., more electropositive). The relative electropositivity of **DAPN·1,3-BBEC** is consistent with inclusion and π–π interactions involving the electron-rich *m*-xylene guests.^24^ We note that the C atoms of the central phenyl ring of the boronic ester display higher electron density within the bridging group. The increase in electron density is in contrast with **1,3-BBEC** as a pure form. Su has reported DFT calculations that address the extent of overlap of B atoms of trigonal-planar geometry with pendant phenyl rings.^25^ We have also reported on similar electronic effects and guest inclusion involving assemblies of single boronic esters.^24^ The approximate perpendicular orientation assumed by **1,8-nap** with the diboron ester moiety is also reminiscent of frameworks used to form molecular and supramolecular dyads.^26^

## Conclusion

The self-assembly of a series of U-shaped bipyridines based on **1,8-nap** with the diboronic ester **1,3-BBEC** has resulted in the formation of novel “plug-in-socket” type architectures. We are now studying the scope of the self-assembly process to other diboronic esters, as well as expanding the host–guest properties. We expect our efforts to contribute to the generation of B-based functional materials.

## Supporting Information Available

General experimental details, synthetic procedures for **DPN·1,3-BBEC**, **DEPN·1,3-BBEC**, and **DAPN·1,3-BBEC**. ^1^H NMR and X-ray diffraction data, and electrostatic potentials for **DPN·1,3-BBEC**, **DEPN·1,3-BBEC**, and **DAPN·1,3-BBEC** (PDF)

## Accession Codes

CCDC 2074336–2074340 contain the supplementary crystallographic data for this paper. These data can be obtained free of charge via www.ccdc.cam.ac.uk/data_request/cif, or by emailing data_request@ccdc.cam.ac.uk, or by contacting The Cambridge Crystallographic Data Centre, 12 Union Road, Cambridge CB2 1EZ, UK; fax: +44 1223 336033.
