# Supplementary material for: Self-Assembly of Diboronic Esters with U-Shaped Bipyridines: “Plug-in-Socket” Assemblies
Source: Cryst Growth Des. 2021 Jul 13;21(8):4482–7. doi: 10.1021/acs.cgd.1c00382 (PMC8411870; doi:10.1021/acs.cgd.1c00382)
Supplement: Supplementary file 1 — cg1c00382_si_001.pdf [file cg1c00382_si_001.pdf]

Supporting Information

Self-Assembly of Diboronic Esters with U-Shaped Bipyridines: ‘Plug-in-Socket’ Assemblies

Christopher J. Hartwick, Shweta P. Yelgaonkar, Eric W. Reinheimer, Gonzalo Campillo-Alvarado, Leonard R. MacGillivray*

Department of Chemistry, University of Iowa, Iowa City, IA, 52242

Content:

1. U-shaped bipyridine syntheses. S2
2. Assembly formation. S3
3. ^{1}H NMR data of assemblies. S4-S6
4. X-ray powder pattern data. S7-S9
5. Molecular modeling S10-S12
6. References S13

1.0 U-shaped bipyridine syntheses.

All materials were obtained from commercial sources and used as received unless indicated. Diiodonaphthalene, catechol, 4-vinyl pyridine, 4-pyridineboronic acid, were purchased from Sigma-Aldrich. Palladium bis-triphenylphosphine dichloride was purchased from Acros. 1,4 phenyldiboronic acid was purchased from Combi-blocks. Potassium carbonate was purchased from Fisher Scientific. Chloroform (99%), toluene (99%), *m*-xylenes (95%) were purchased from Sigma-Aldrich.

DPN. Yield: 33.1%, gray crystals, 1:2 EtAc /*n*- hexanes. ^{1}H NMR agrees with reported values.[1]

DEPN. Literature procedure was followed. Yield: 35.2%, Amber-yellow crystals. ^{1}H NMR agrees with reported values.[2]

DAPN. From the literature, procedure was followed. Brown solid, Yield: 63.5%.[3] ^{1}H NMR agrees with reported values.

2.0 Assembly formation.

DPN·(1,3-BBEC). To a 30 ml scintillation vial, **DPN** (0.011 grams, 0.04 mmols), 1,3-phenyldiboronic acid (0.007 grams, 0.04 mmols), and catechol (0.008 grams, 0.08 mmols) were added along with 5 ml of chloroform after grinding with a mortar-and-pestle. The grinding apparatus was rinsed with 4 ml (2 x 2 ml) of chloroform which was added to the vial along with 2ml of toluene which was subsequently heated until a homogenous solution was obtained. Upon cooling, the top of the vial was left open and solvent was allowed to evaporate, producing translucent gray crystals within 48 hours.

^{1}H NMR (400 MHz, CD_2Cl_2): δ 8.58-8.60 (4H, dd, J = 5.3, 1.3 Hz, Py-H_d), 8.20-8.22 (2H, dd, J = 8.3, 1.1 Hz, Aryldiboronic-H_j), 7.74-7.78 (2H, dd, J = 8.2, 7.2 Hz, Naphth-H_b), 7.65-7.66 (2H, dd, J = 7.3, 1.4 Hz, Naphth-H_a), 7.53-7.55 (2H, dd, J = 7.2, 1.2 Hz, Naphth-H_c), 7.45-7.47 (4H, dd, J = 5.3, 1.3 Hz, Py-H_e), 7.35-7.38 (1H, t, J = 3.4 Hz Aryldiboronic-H_i), 7.36 (0.9H – Included solvent), s, Chloroform- H), 6.87-6.89 (1H, m, , J = 6.0, 3.5 Hz Aryldiboronic- H_f), 6.85-6.86 (4H, dd, J = 5.6, 3.4 Hz, Catechol- H_g), 6.71-6.73 (4H, m, Catechol-H_h).

DAPN·(1,3-BBEC). To a 30ml scintillation vial, **DAPN** (0.031 grams, 0.09 mmols), 1,3 phenyldiboronic acid (0.015 grams, 0.09 mmols), and catechol (0.02 grams, 0.18 mmols) were added along with 5 ml of chloroform after grinding with a mortar-and-pestle. The grinding apparatus was rinsed with 4 ml (2 x 2ml) of chloroform which was added to the vial along with 2 ml of *m*-xylenes which was subsequently heated until a homogenous solution was obtained. Upon cooling the top of the vial was left open and solvent allowed to evaporate, producing crystals within 48 hours.

^{1}H NMR (400 MHz, CD_2Cl_2) δ 8.57 (4H, d, J = 6.6 Hz, Py-H_d), 8.05 (s, 2H, Aryldiboronic-H_b), 7.99 (2H,d, J = 8.5 Hz, Aryldiboronic-H), 7.72 (2H, d, J = 8.6 Hz, Naphth-H_c), 7.62 (2H, d, J = 7.3 Hz, Naphth-H_a), 7.56 – 7.54 (4H, m, Py-H_e), 7.38 (1H, t, J = 7.3 Hz, Aryldiboronic-H_i), 7.16 (2H, t, J = 7.5 Hz, Aryldiboronic-H_f), 7.04 (2H, s, m-xylenes), 6.99 (4H, d, J = 9.7 Hz, m-xylenes), 6.89 (d, J = 9.0 Hz, Catechol- H_g), 6.76 (4H, d, J = 9.0 Hz, Catechol-H_h), 2.34 (s, 12H, m-xylenes).

DEPN·(1,3-BBEC). To a 30ml scintillation vial, **DEPN** (0.03 grams, 0.09 mmols), 1,3 phenyldiboronic acid (0.015 grams, 0.09 mmols), and catechol (0.02 grams, 0.18 mmols) were added along with 5 ml of chloroform after grinding with a mortar-and-pestle. The grinding apparatus was rinsed with 4 ml (2 x 2ml) of chloroform which was added to the vial along with 2 ml of toluene which was subsequently heated until a homogenous solution was obtained. Upon cooling the top of the vial was left open and solvent allowed to evaporate, producing crystals within 48 hours.

^{1}H NMR (400 MHz, CD_2Cl_2) δ 8.56 (4h,d, J = 6.7 Hz, Pyr-H_f), 8.15 (2H, d, J = 16.0 Hz, Alkene-H_e), 7.99 (2H, d, J = 8.2 Hz, Aryldiboronic-H_m), 7.74 (2H, d, J = 3.2 Hz, Naphth-H_b), 7.72 (2H, d, J = 3.3 Hz, Naphth-H_a), 7.63 – 7.59 (2H, m, Naphth-H_c), 7.50 (4H, d, J = 6.8 Hz, Pyr-H_g), 7.37 (2H, m, Aryldiboronic-H_l), 7.00 (2H, d, J = 16.0 Hz, Alkene-H_d), 6.89 (1H, s, Aryldiboronic- H_i), 6.89 – 6.84 (4H,m, Catechol-H_j), 6.75 (4H, m, Catechol- H_k).

3.0 ^{1}H NMR data of assemblies.

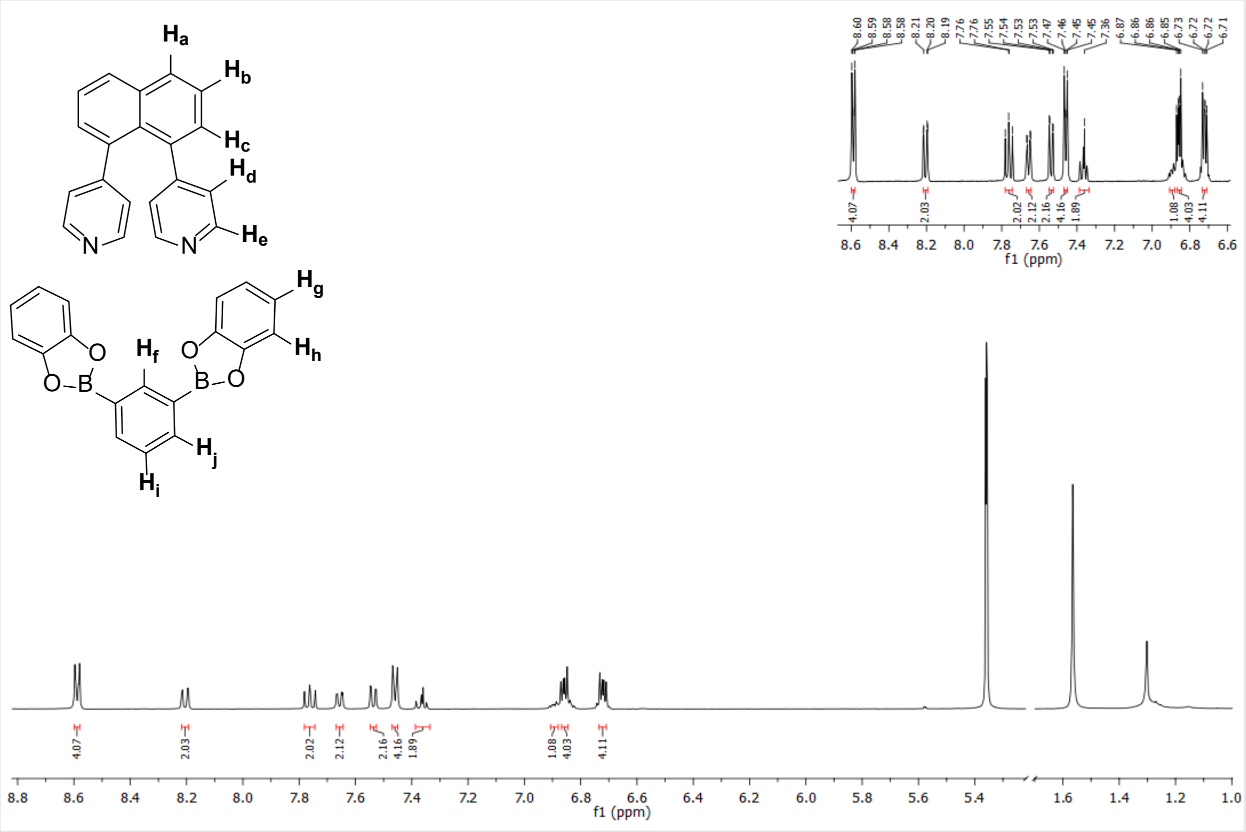

Figure S1. ^{1}H NMR spectrum of **DPN**·(**1,3-BBEC**)·$CHCl_3$.

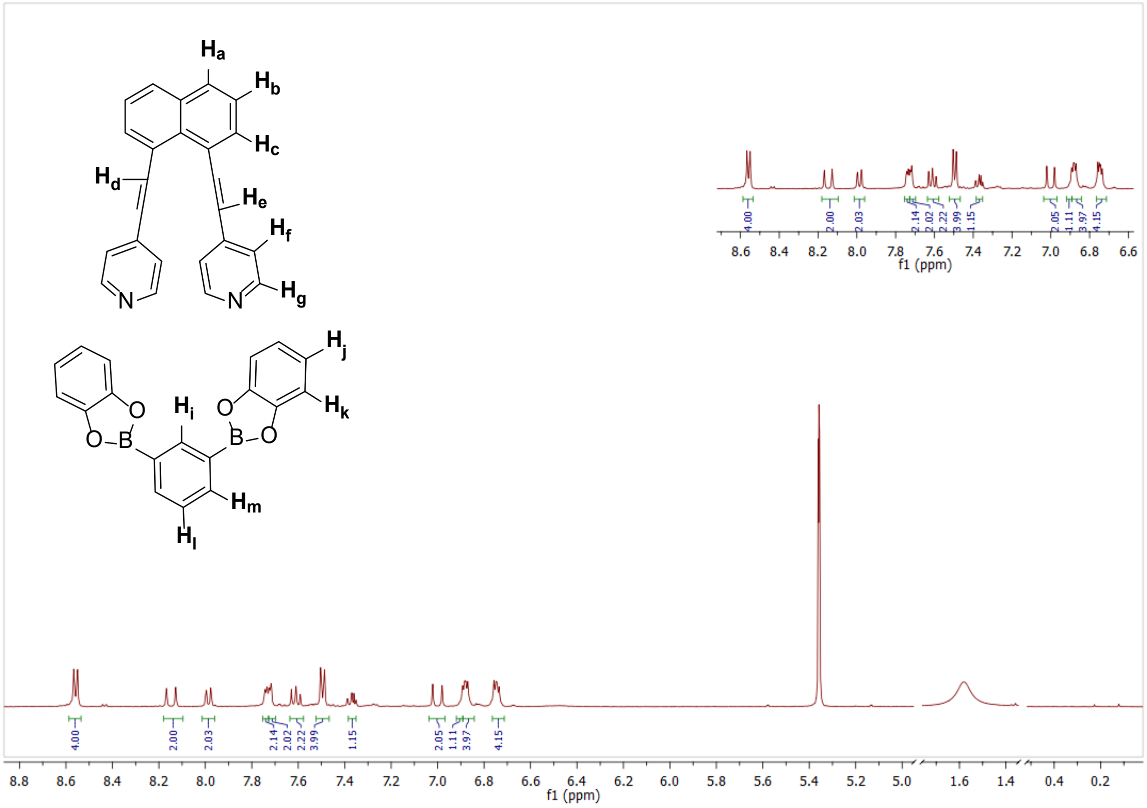

Figure S3. ^{1}H NMR spectrum of **DEPN**·(**1,3-BBEC**).

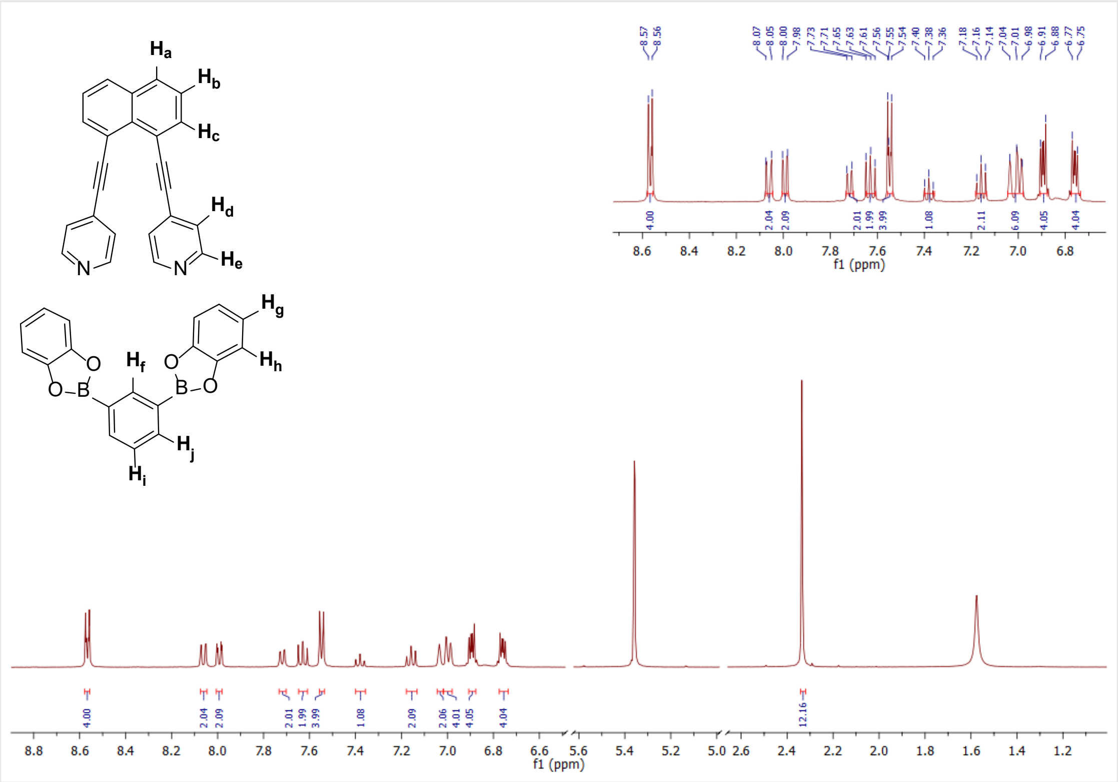

Figure S3. ^{1}H NMR spectrum of **DAPN**·(**1,3-BBEC**)·2(***m*-xylene**).

4.0 X-ray powder pattern data.

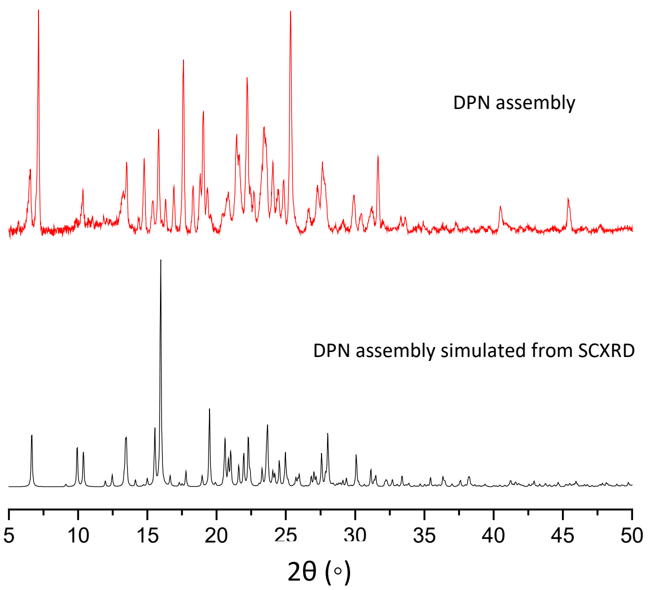

Figure S4. Experimental and simulated PXRD of **DPN**·(**1,3-BBEC**)·$CHCl_3$.

Note: Crystal degradation occurred due to loss of included $CHCl_3$.

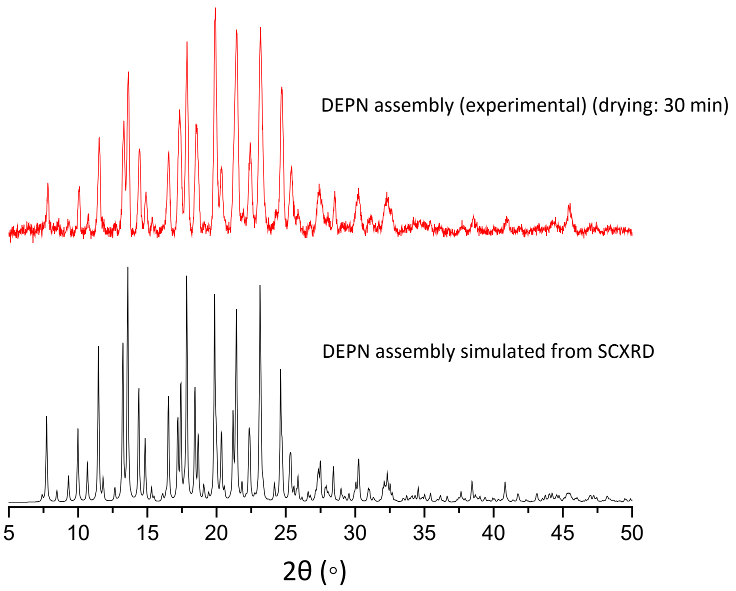

Figure S5. Experimental and simulated PXRD patterns of **DEPN**·(**1,3-BBEC**).

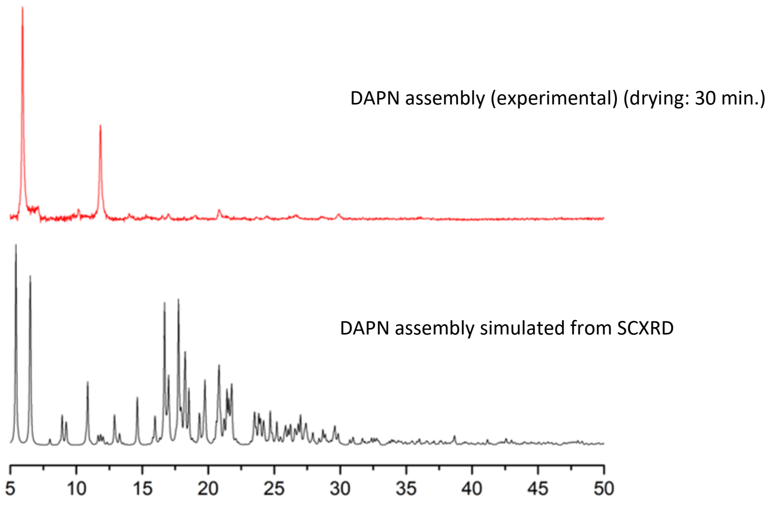

Figure S6. Experimental and simulated PXRD patterns of **DAPN**·(**1,3-BBEC**)·2(***m*-xylene**).

Note: Crystal degradation occurred due to loss of included *m*-xylene.

5.0 Molecular Modeling

Electrostatic potential maps were generated for each **1,8-nap** complex by calculations at ground state in gas phase using Spartan ’18 V1.2.0 software. Calculations were performed with B3LYP/6-31G* using the density functional model in a vacuum. Charge value ranges were set to a standard range of +/- 200 KJ/mole with isovalues of 0.002 for each. Single-crystal lattice data was used as a starting point to conduct single-point energy calculations.

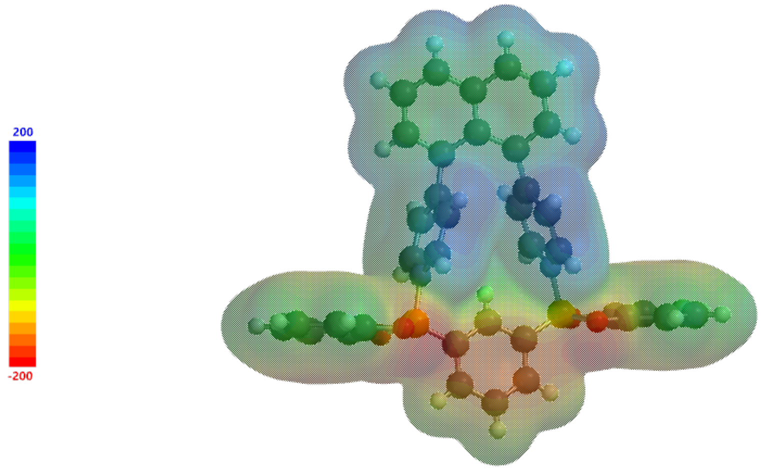

Figure S7. Electrostatic potential map of **DPN**·(**1,3-BBEC**).

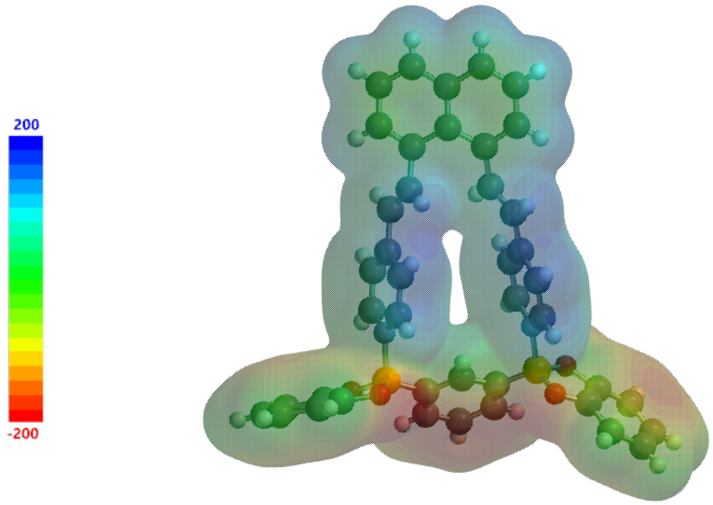

Figure S8. Electrostatic potential map of **DEPN**·(**1,3-BBEC**).

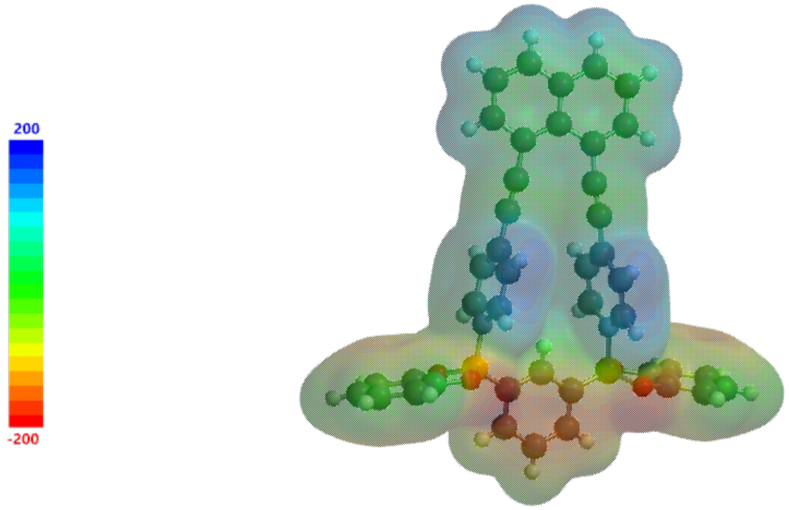

Figure S9. Electrostatic potential map of **DAPN**·(**1,3-BBEC**).

6.0 References

1. Sinnwell, M. A.; Ingenthron, B. J.; Groeneman, R. H.; MacGillivray, L. R., Stereoselective and quantitative [2+2] photodimerization of a symmetrical octafluoro stilbene in the solid state: Face-to-face stacking of the fluorinated rings in trans-1,2-bis(2,3,5,6-tetrafluorophenyl)ethylene. *J. Fluor. Chem.* **2016**, *188*, 5-9.
2. Laird, R. C.; Sinnwell, M. A.; Nguyen, N. P.; Swenson, D. C.; Mariappan, S. V.; MacGillivray, L. R., Intramolecular [2+2] Photodimerization Achieved in the Solid State via Coordination-Driven Self-Assembly. *Org Lett* **2015,** *17*, 3233-3235.
3. Yelgaonkar, S. P.; Kiani, D.; Baltrusaitis, J.; MacGillivray, L. R., Superstructural diversity in salt-cocrystals: higher-order hydrogen-bonded assemblies formed using U-shaped dications and with assistance of pi(-)-pi stacking. *Chem. Commun.* **2020,** *56*, 6708-6710.
